# Overview of technical solutions and assessment of clinical usefulness of capsule endoscopy

**DOI:** 10.1186/s12938-015-0108-3

**Published:** 2015-12-01

**Authors:** Robert Koprowski

**Affiliations:** Department of Biomedical Computer Systems, Faculty of Computer Science and Materials Science, Institute of Computer Science, University of Silesia, ul. Będzińska 39, 41-200 Sosnowiec, Poland

**Keywords:** Biomedical, Capsule, CCD, CMOS, Image processing, Medical image, PillCam, SB2

## Abstract

The paper presents an overview of endoscopic capsules with particular emphasis on technical aspects. It indicates common problems in capsule endoscopy such as: (1) limited wireless communication (2) the use of capsule endoscopy in the case of partial patency of the gastrointestinal tract, (3) limited imaging area, (4) external capsule control limitations. It also presents the prospects of capsule endoscopy, the most recent technical solutions for biopsy and the mobility of the capsule in the gastrointestinal tract. The paper shows the possibilities of increasing clinical usefulness of capsule endoscopy resulting from technological limitations. Attention has also been paid to the current role of capsule endoscopy in screening tests and the limitations of its effectiveness. The paper includes the author’s recommendations concerning the direction of further research and the possibility of enhancing the scope of capsule endoscopy.

## Background

Capsule endoscopy is a technique known for many years which enables to watch the mucosa of the small intestine, large intestine and oesophageal. It involves swallowing a capsule and on-line or off-line data transmission to a receiver or computer. Using this technique, it is possible to access the small intestine and diagnose different types of diseases which are difficult to diagnose by means of other methods (classic endoscopy with the use of a flexible tube). These diseases include: (1) chronic gastrointestinal bleeding; (2) unexplained iron deficiency anaemia; (3) Crohn’s disease; (4) tumour of the small intestine; (5) damage to the mucosa of the small intestine; (6) celiac disease; (7) gastrointestinal polyposis syndromes. Moreover, there are few restrictions on the use of capsule endoscopy. Contraindications include: (1) narrowing and obstruction of the gastrointestinal tract; (2) dysphagia; (3) disorders of gastrointestinal motility; (4) intestinal fistula; (5) multiple or large gastrointestinal diverticula; (6) abdominal surgeries; (7) pregnancy; (8) a heart pacemaker. With so few contraindications, gastrointestinal diagnostics using capsule endoscopy is very popular and, as a result, it is being rapidly developed. This is evidenced by a number of research papers published worldwide. The details of these papers are described in the following subsections.

### State of the art associated with endoscopy: a review of articles

At the time of writing this paper there were 4234 articles containing the word “Capsule Endoscopy” according to the data published on AuthorMapper. A similar number of articles are available on PubMed—3488 articles. The number of articles published per country is shown in Table 1. It should be emphasized that the number of papers given on AuthorMapper is sometimes inflated by multiple counting of the same work. Thematic areas with the greatest number of articles are: Medicine & Public Health—3201 articles (e.g. [36]), Gastroenterology—1608 articles (e.g. [37]), Hepatology—875 articles ([38]), Oncology and Surgery—763 and 744 articles respectively ([39, 40]). As shown in Table 1 and above, the area of research devoted to capsule endoscopy is essentially associated with medicine. The known review articles related to capsule endoscopy and its use in medicine also deal with this area. For example, these are works [31] or [32]. In the Web of Science database it is possible to trace publications which have the largest number of citations or are the most influential. The first authors with the greatest number of citations are: Iddan [41] 1101 citations, Costamagna [42] 557 citations, Pennazio [43] 510 citations and then Ell C. [44] and Lewis [45] 471 and 360 citations respectively. The first mentioned author, Iddan G, is the inventor of capsule endoscopy. He was inspired by the 1966 film “Fantastic Voyage” about a trip to the interior of the body. The other authors present new approaches to the use of capsule endoscopy.

**Table 1:** Comparison of the number of articles containing the word “capsule endoscopy” per countries, institutions and authors as at 06.02.2015 (only the first five are provided)

Characteristic	Units/authors	Number of articles	Examples
Country	United States	1484	[1–3]
	Germany	323	[4–6]
	Japan	322	[7–9]
	Italy	297	[10–12]
	United Kingdom	241	[13–15]
Institution	Columbia University	364	[16–18]
	Mayo Clinic	49	[3, 19–21]
	University of Amsterdam	26	[22–24]
	Kyushu University	23	[25–27]
	Harvard Medical School	21	[28–30]
Author	Minc N.	358	[31]
	Packer C.	46	[32]
	Dijk C.	19	[33]
	Lui T. H.	15	[34]
	Wang Z.	15	[35]

The information given in Table 1 and above summarizes the state of capsule endoscopy in the world. The thematic division of articles on capsule endoscopy where the area of medicine and public health comes first is particularly noteworthy. Moreover, it should be noted that the areas of microbiotics or computer science, necessary to program the internal capsule control, are not present in the tables. The areas of hepatology and oncology, in which 875 and 763 articles respectively are on the use of capsule endoscopy, are also of interest. The authors listed above deal with biomedical engineering (Iddan G.) and medical diagnostics (Costamagna G., Pennazio M., Ell C., Lewis B.). The direction of research that largely focuses on clinical applications and to a lesser extent (almost residual) relates to new developments and electro-technical [46, 47] modifications of endoscopic capsules is equally interesting. The consequence is also a limited number of citations of articles related to technical descriptions of new solutions.

The works on micro-robotics are slightly different, and also the number of granted patents for capsule endoscopy differs. In the database of google patents there are 3530 patent descriptions containing the word “capsule endoscopy”. However, most of the solutions require additional equipment (data capture systems or systems controlling the capsule route) and not individual innovative capsule solutions. Looking at both medicine and engineering, it seems that the former one is developing very well in terms of various types of applications and diagnostics using capsules. The majority of the afore-mentioned publications are devoted to this area. The technological development of endoscopic capsules looks far worse. Therefore this paper is devoted to the review of the state of the art with particular emphasis on practical clinical usefulness.

### Nomenclature used in the field of capsule endoscopy

Particular attention should be paid here to the following distinction between five terms known from literature:

- VCE: video capsule endoscopy—capsule endoscopy including an imaging device such as a CCD (not relating to endoscopic capsules devoid of an image sensor, the capsule does not have to be wireless);
- WVE: wireless video endoscopy (not necessarily a capsule);
- CE: capsule endoscopy—endoscopic capsule (not necessarily wireless);
- WCE: wireless capsule endoscopy (not necessarily containing an image sensor);
- WVC: wireless video capsule.

**Fig. 1:**
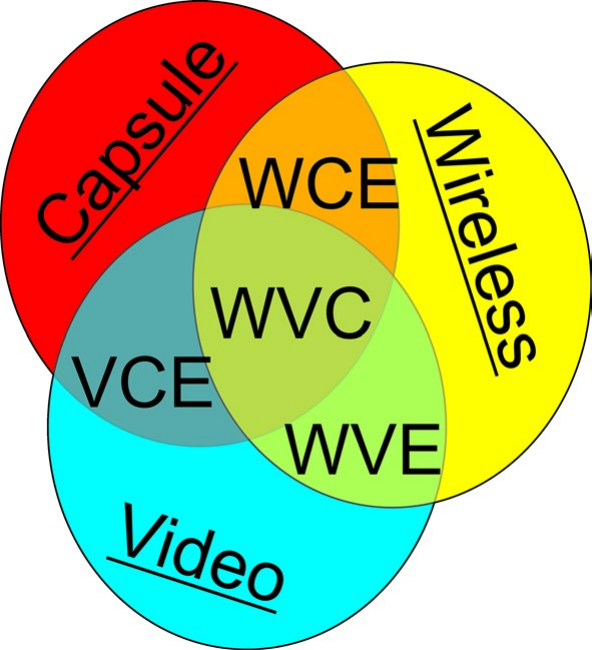
Distinction of the nomenclature relating to capsules, wirelessness and video. *Red* shows the area covered by the definition “Capsule”, *yellow* “Wireless” and *blue* “Video”. The respective intersections of these areas (*circles*) refer to different types of endoscopy. WCE (video capsule endoscopy), VCE (wireless capsule endoscopy), WVE (wireless video endoscopy) when two areas intersect and WVC (wireless video capsule) when all three areas intersect

The distinction is presented in Fig. 1. In literature these terms are often confused and used interchangeably. Consequently, in this paper, they will be all referred to as “capsule endoscopy”.

Typical known endoscopic capsules that are currently used in medical diagnosis are presented below. The following section presents new types of endoscopic capsules representing the prospects and direction of development in the coming years.

## Typical, most common capsule solutions (standard techniques)

**Fig. 2:**
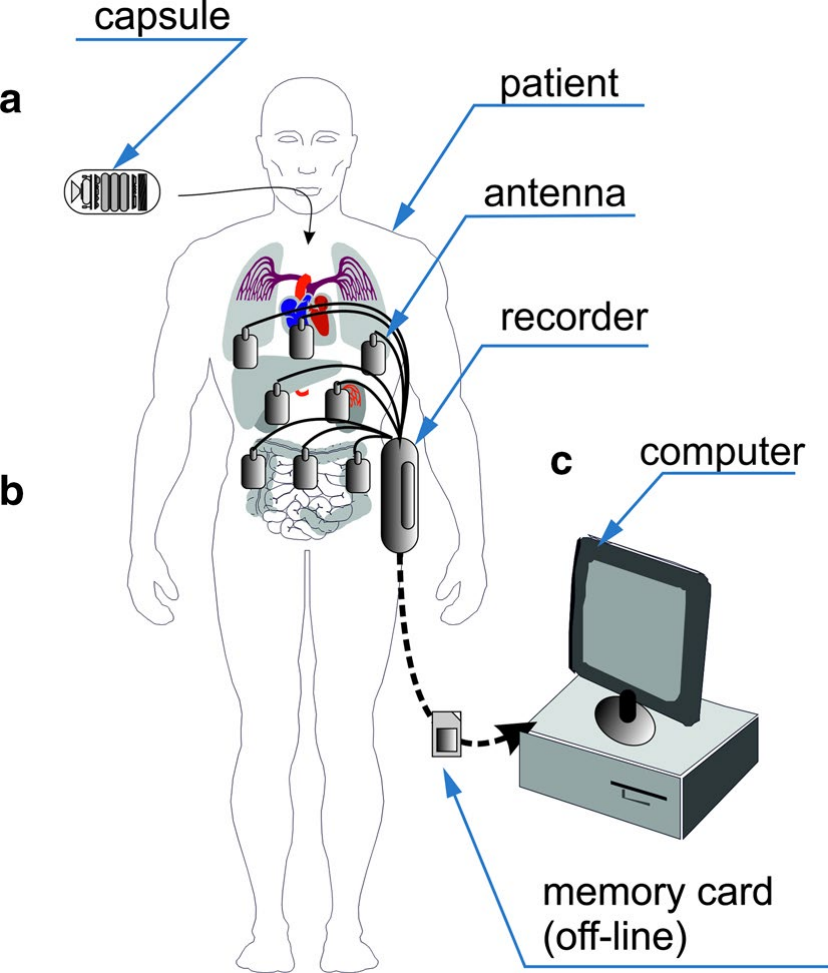
Basic capsule endoscopy system. It consists of **a** a capsule, **b** a recorder with antennas placed at the patient’s side, and **c** a computer with software for data analysis. This is a typical, most popular set of capsule endoscopy

**Fig. 3:**
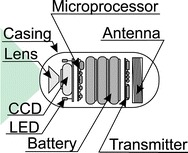
Block diagram of a typical endoscopic capsule. The capsule contains: a lens with CCD, a set of LEDs illuminating the test area, microprocessor control, transmitter with antenna and power supply in the form of batteries

The most popular capsule endoscopy systems consist of three elements: (a) a capsule swallowed by the patient; (b) a system for acquisition of wirelessly sent data; (c) a computer with software for analysing the recorded data. Details of the system are shown in Fig. 2. The system shown in Fig. 2 is a well-known capsule endoscopy system proposed by Iddan [41] and used to date. Its single elements evolve in line with advances in miniaturization in electronics and micro-robotics, finding more areas of clinical applications. The most popular endoscopic capsules along with characterization of the acquisition systems (number of antennas) are shown in Table 2. As is clear from the data provided in the summary in Table 2, capsules are divided in terms of their application and they can be used to diagnose: (1) the small intestine; (2) the large intestine; (3) the oesophagus. Depending on the application, they have different operating time, frequency of taking images and number of cameras. In the oesophagus diagnosis, two cameras, which are disposed on opposite sides of the capsule, are used due to the relatively short residence time of the capsule in the oesophagus, a high frequency of taking oesophagus images and short operating time. In the diagnosis of the small and large intestine, one camera and not too high frame rate are used (due to the need to save battery—the average residence time of the capsule in the small and large intestine is approximately 8 h). In addition to the camera (cameras), typical capsules also contain other electronic components necessary for proper operation—Fig. 3. A typical endoscopic capsule consists of: a CCD or a CMOS camera placed on its one or both sides providing images with a typical resolution of 320 × 240 pixels, a lens allowing for wide-field acquisitions (170°) of the visible intestine area, from 4 to 8 LEDs illuminating the imaging area in visible light, a battery consisting of two 1.5 V cells, a transmitter with an antenna that allows wireless communication with a receiver placed on the patient (Fig. 2). For a typical endoscopic capsule, the operating time is a few hours and significantly exceeds the needs—i.e. the average residence time of the capsule in the body. In practice, namely in clinical applications, there are two important aspects in these typical solutions: (1) battery life; (2) maximum distance of data transmission. *In the first case*, situations when capsules get stuck in the intestine and the possibility of observing the reasons for these situations are clinically vital. *In the latter case*, the convenience of the patient who has to wear the apparatus receiving the data sent by the capsule is important. A typical maximum distance of the receiver from the capsule is a few centimetres (for typical data transmission in the band 434 MHz with a 267 kb/s data rate). Data are transmitted unidirectionally (from the capsule to the receiver). After the acquisition, the storage medium (usually a memory card) is transferred manually to a computer where the data (images) are analysed using dedicated software. Typical software (e.g. CapsoView™ CVV is CapsoVision’s ver 1.0) enables to view the sequence of images stored in the form of a video, mark the selected pathological area and create reports. Clinically, the described known solutions of a capsule endoscopy system have drawbacks. These are:

**Table 2:** Summary of the most popular endoscopic capsules and their basic parameters

	Given imaging—Israel			KIST—Korea	Olympus—Japan	Jinshan—China	RF System Lab.—Japan	
	Pillcam SB2	Pillcam eso	Pillcam colon	Mirocam	Endocapsule	OMOM	Norika 3	Sayaka
Length (mm)	26	26	32	24	26	27.9	23	23
Diameter (mm)	11	11	11.6	11	11	13	9	9
Weight (g)	3.4	3.4	3.4	3.4	3.8	6	–	–
Number of cameras	1	2	2	1	1	1	1	1
Number of frames per second	2	18	4-35	3	2	2	30	30
Sensor	CMOS	CMOS	CMOS	CCD	CCD	CCD	CCD	CCD
Operation time (h)	8	20 m	10	11	9	8	8	8
Number of antennas	8	3	8	9	8	14	8	8
Snooze option	No	No	Yes	No	No	No	Yes	Yes
Destination—the test area	Small (S) intestine	Esophagus	Large (L) intestine	S/L intestine	S intestine	S intestine	S intestine	S intestine

- ensuring the patency of the gastrointestinal tract;
- wireless communication limitations;
- the ability to control the capsule position;
- a change of the imaging area;
- a reduction in the length of the capsule endoscopic video watched by a doctor.

For these reasons, capsule endoscopy technology is still being modified and improved. These drawbacks have been offset by using the following solutions.

### Ensuring the patency of the gastrointestinal tract

In order to verify the patency of the gastrointestinal tract, a pilot capsule (Agile) is used for a typical size of the endoscopic capsule. This capsule has a typical size (26 × 11 mm) and dissolves in the intestine after 30 h if it gets stuck. The pilot capsule in the previous version contained a thin marker (2 mm in diameter) and in the newer one a RFID (radio-frequency identification) tag of the same diameter. The pilot capsule, however, increases the total examination cost and extends the time of obtaining diagnostically relevant results.

### Limitations of wireless communication

**Fig. 4:**
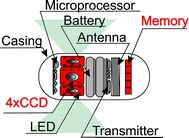
CapsoCam SV-1—block diagram. *Red* indicates changes compared to the original version of the capsule. There are four CCD cameras placed relative to each other at an angle of 90^°^ and a data memory

Regardless of the technological development, wireless data transmission still entails limitations. Therefore, in 2013 CapsoVision introduced the CapsoCam SV-1 shown in Fig. 4. The capsule presented in Fig. 4 has the following parameters: (1) viewing area—360^o^; (2) the number of frames per second—20, (3) operating time—15 h; (4) data record—flash memory; (5) the size of 31 × 11 mm; (6) LED lighting -16; (7) the number of cameras—4; (8) motion sensor and LED brightness. Writing data directly in the flash memory has two drawbacks: (1) clinical—no possibility of observing the area being imaged on-line, (2) technical—the need for a special docking station that enables to access the memory and read data. The flash memory is one of the biggest advantages of this capsule allowing for complete elimination of the receiver and the need to transfer data wirelessly. In addition, this type of capsule has four cameras placed at an angle of 90° relative to each other. This increases the area and fidelity of imaging—cameras are often arranged at right angles relative to the imaged area.

### Controlling the capsule position

**Fig. 5:**
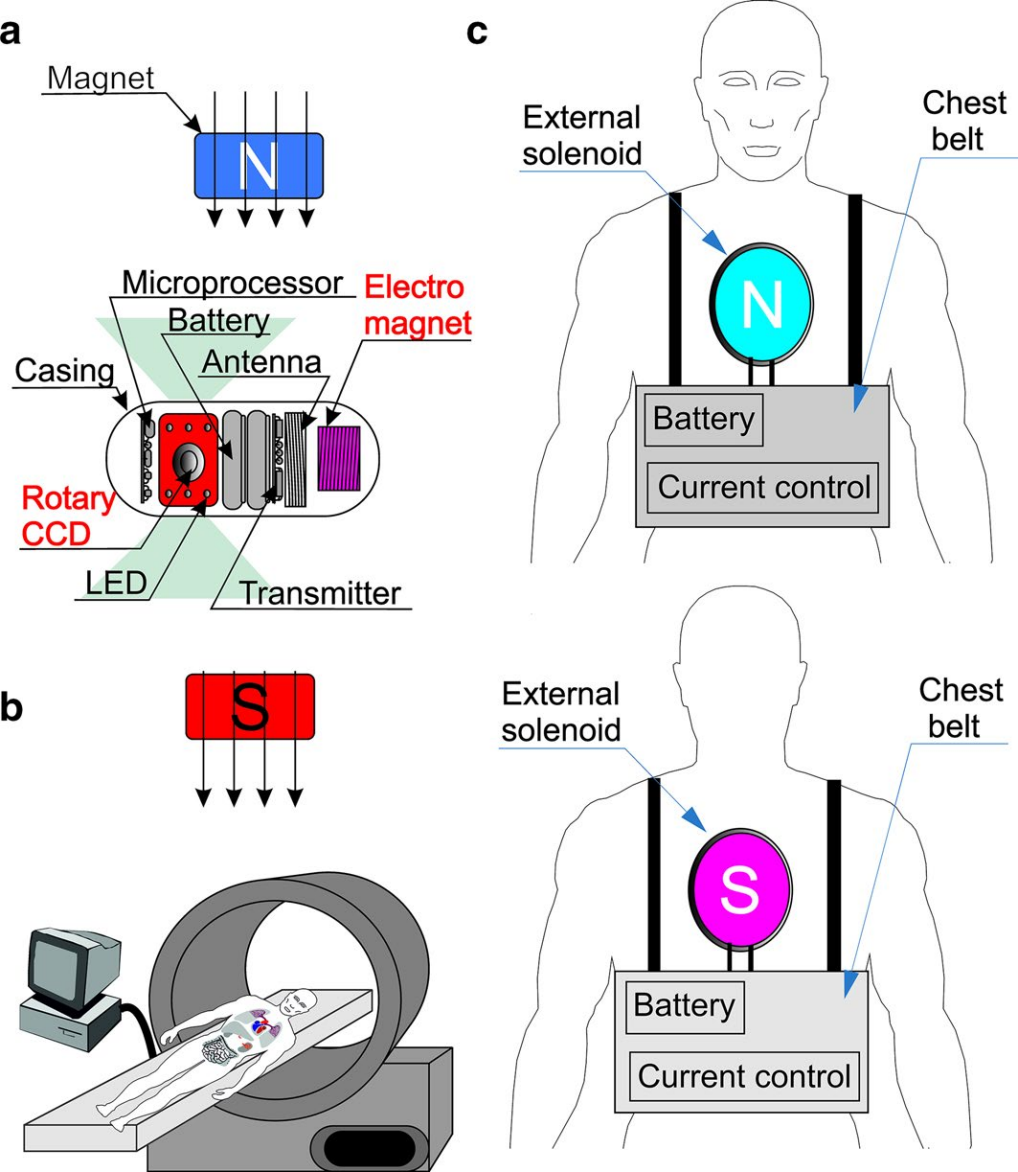
Sayaka capsule by RF System Lab with internal control with the use of external and internal constant magnetic fields placed inside the capsule, **a** 3D electromagnet (with a power of 300 mW, 125 kHz operating frequency, and the size of 10 × 10 × 10 mm) **b** an external stationary electromagnetic field and **c** a portable external set of magnetic field in accordance with paper [48]

Controlling the position of the capsule is very important clinically because of the need for orientation and careful observation of characteristic, diagnostically interesting areas. This is done by using an electromagnetic field. The capsule has a permanent magnet and the external magnetic field is changed, or vice versa—Fig. 5. The coils of electromagnet placed inside the capsule (Fig. 5) are in three axes so that the capsule can perform virtually any motion. Despite the full freedom of movement (front/rear, left/right, up/down, rotation, side, attitude, jump), a diagnostician most often uses rotation [49]. This is due to the need for accurate viewing of the selected, diagnostically interesting area. In practice, however, the patented solution of the electromagnetic device placed on the patient [49] has not gained a foothold. To control the capsule, the same technique yet stationary is used [50–54], for example, the so-called MGCE system which is the result of collaboration between Siemens Healthcare and Olympus Medical Systems Corporation. An electromagnetic assembly is in the form of a cylindrical roller inside which a patient is placed in the supine position—as in the case of tomography.

### Change of the imagining area

**Fig. 6:**
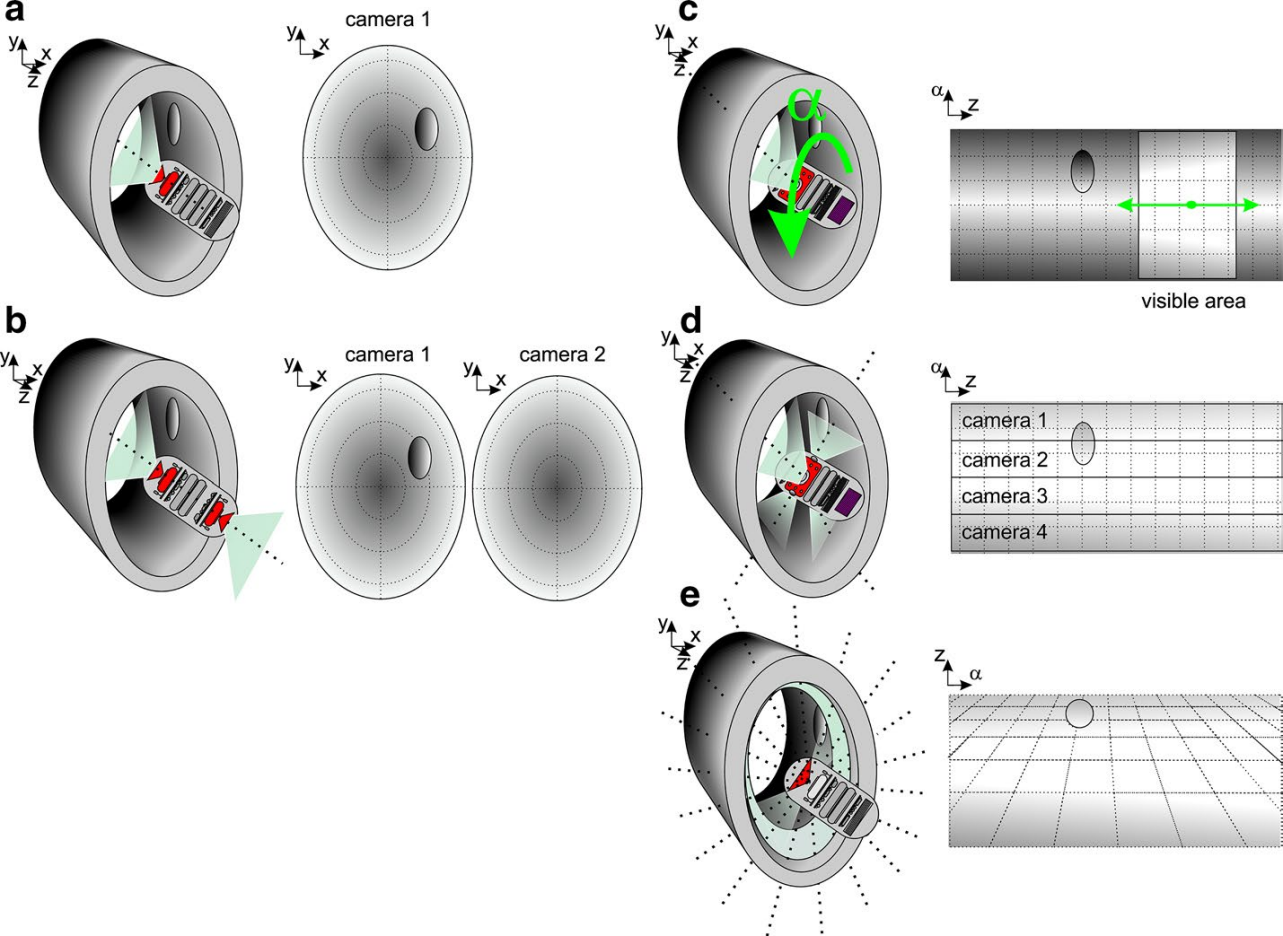
Ways of deploying cameras in capsules: **a** one camera placed in the main axis of the capsule (Pill Cam SB); **b** two cameras placed in the main axis of the capsule (Pill Cam COLON); **c** one rotatable camera positioned perpendicular to the main axis of the capsule (e.g. Sayaka RF System Lab); **d** four cameras arranged perpendicular to the main axis of the capsule (e.g. CapsoCam SV-1); **e** one camera placed in the main axis of the capsule with a conical mirror (RICE—National Chiao Tung University-[62])

The imaging area is very important in three-dimensional reconstruction, determination of the exact position of the capsule and, above all, determination of the size of visible objects (polyps, detachment, bowel perforation). For a typical camera position (Fig. 3), the imaging area in front of the capsule comprises an angle of about 170^°^ [55–58]. The angle is closely dependent on the lens and the casing type. However, the problem here is the accurate determination of the size of the observed objects because of the perspective—an unknown object distance from the camera. 3D and 4D reconstructions are performed [59–61] on the basis of the approximate information about the capsule movement speed, the positions of individual objects in the mage as well as changes and sizes along with the capsule movement. An alternative method is to place a rotatable camera or group of cameras transversely to the main axis of the capsule (Fig. 5a). Another way is to place capsules containing a conical mirror in the main axis, which allows for simultaneous observation of the full circumference of the intestine portion. All known types of deployment of cameras in capsules are shown in Fig. 6. The methods of image analysis and processing provide correct results in segmentation of specific areas such as the areas of bleeding. However, significant limitations directly associated with the place of imaging relate to the aspects of metrology. The greatest errors of up to several tens of percent are obtained during the assessment of the bleeding area size in capsules with a camera placed in their main axis (Fig. 6 a, b—Pill Cam SB/Colon). In the solution shown in Fig. 6c (Sayaka RF System Lab), the main problem is the dynamic error associated with the helical nature of imaging of specific portions of the intestine slice. The smallest acquisition errors (both dynamic and static ones) are obtained for the imaging methods shown in Fig. 6d, e. A few percent error values are for the solution d (CapsoCam SV-1). They are associated with the differences between CCDs of the four cameras. However, in the case of e) (Fig. 6e—RICE—National Chiao Tung University-[62]), they are associated with the non-axial embodiment of the conical mirror.

### Reducing the length of the capsule endoscopic video watched by a doctor

A typical video from the capsule endoscopy examination lasts a few hours. Diagnosticians must watch the entire video to make a diagnosis because in typical clinical cases there is no indication in which bowel area they are to search for bleeding, tumours or other types of damage caused, for example, by non-steroidal anti-inflammatory drugs (NSAIDs) or radiotherapy. The capsule movements result from the impact of gravity and bowel movements. In the small intestine there are two types of contractions: (1) peristaltic and (2) staple (segment). The first type of contractions is responsible for the transit of food (relatively linear in nature), while the latter one is responsible for mixing food (rather chaotic). These movements are regularly punctuated by moments of relaxation in the intestinal muscles. The result is that the image obtained from capsule endoscopy is highly diverse—moments of stillness, the camera obscured by food debris, moments of dynamic movements and thereby changes of the imaging area. Therefore the video watched by a diagnostician requires acceleration, deceleration, and sometimes it must be watched frame by frame. Consequently, there is an ongoing research study related to the implementation of image analysis and processing methods allowing for automatic video analysis. The use of, for example, a model deformed for camera motion analysis and a universal method of feature selection and classification results in a much shorter video. In comparison to this, commercial software, for example, RapidReader (by GivenImaging) or free CapView, enables only to find the area of bleeding [63, 64]. Clinically, image analysis and processing such as the selection of image features [65, 66], colour-based segmentation [67], segmentation using vector classifiers and hidden markov models [68], discriminant analysis [69, 70] and others [71–74] allow for a significant reduction in analysis time. Moreover, mathematical morphology methods such as erosion, dilation, opening or closing as well as classical methods of binarization or wavelet analysis can be applied here. Particularly good segmentation results are also obtained for the HSV colour model which allows for precise determination and segmentation of the bleeding areas. According to publications [63–74] the video is shortened by more than 90 %. In clinical practice it means that medical diagnosticians watch a few minutes of the video including all the pathologies detected by the software.

Additional information stored in the memory is extremely useful in the evaluation and interpretation of the video. This is, for example, the current time which is saved for each video frame. Additionally, the patient is often asked to record abnormal situations related to the manual tasks performed during the test (i.e. sudden getting up, running or moving by means of some vehicle). These elements greatly improve interpretation of individual video frames and make it easier to identify a diagnostically interesting video fragment. In addition, in some systems there is the possibility to track the capsule position on the basis of the signal strength and by placing antennas on the patient [63–69].

According to the current state of knowledge, it seems that everything has been done. However, there are many interesting and clinically recognized areas such as the thematic area of biopsy, capsule movement control or the assessment of the possibility of doing surgery with the use of capsules. This is discussed in the next section.

## Prospects and expectations of capsule endoscopy

The previous section presented typical standard techniques and endoscopic capsules commonly available in medical units. The new research area related to capsule endoscopy concerns two areas: (1) biopsy performance and (2) control of the capsule direction and speed of movement. In this area there are many interesting solutions which in the future can be successfully used on an everyday basis. Below there is an overview of this type of solutions indicating their advantages and disadvantages.

### Performance of biopsy

**Fig. 7:**
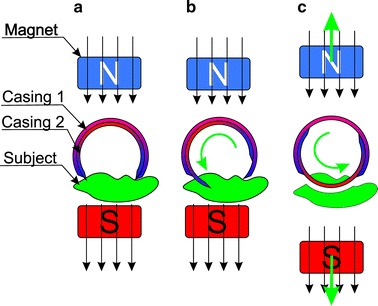
Use of an external magnetic field to bring the cutting tool closer to the biopsy site (**a**), the method of performing biopsy (**b**) and protecting the biopsy material (**c**). Rotation of the two magnetized parts of the capsule takes place after the disappearance of an external magnetic field. The biopsy material is also secured inside the capsule. After leaving the human body, the sample may be further analysed, for example, microscopically/microbiologically

**Fig. 8:**
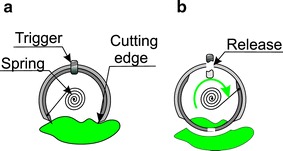
Method for sampling of the intestine: **a** open chamber, strained spring, **b** releasing the spring with simultaneous sampling and storing the sample in the chamber. This solution enables to perform biopsy of a material (e.g. polyp) and secure it inside the capsule

**Fig. 9:**
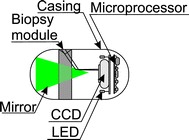
Block diagram of the capsule containing a conical mirror (*green*) placed in its main axis and the biopsy module. The site of biopsy is directly visible to the camera. It is one of the biggest advantages of this solution (the possibility of simultaneous biopsy and observation of this portion in the camera)

**Fig. 10:**
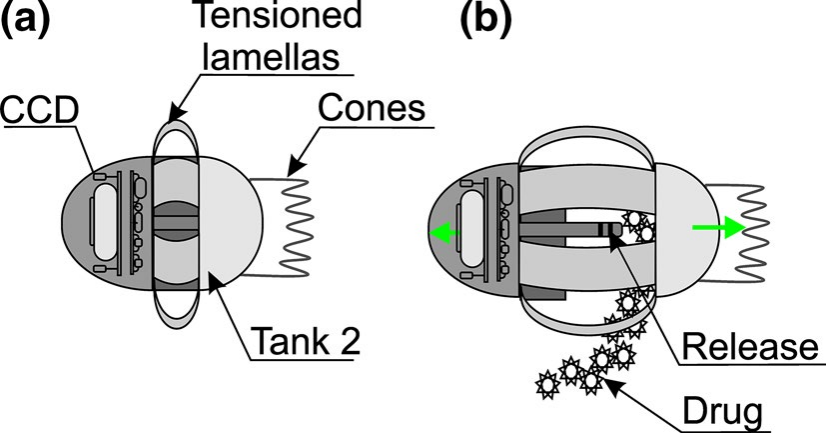
Block diagram of the capsule containing a tank from which micro grippers are emptied. Each micro gripper is made of shape memory metal and tightens taking a slice. **a** A closed tank, **b** an open tank—emptying micro grippers

Biopsy performance is associated with solving four issues: (1) the site of biopsy; (2) bringing the knife (cutting equipment) closer to the located site; (3) biopsy performance; (4) protection of the biopsy material. Location of the biopsy site is performed using a typical camera intended for the intestine diagnosis. Bringing the cutting knife closer is usually performed [75] using an external electromagnetic field—Fig. 7. Figure 7 shows cross-sections of two cylindrical magnetized rings. Without an external magnetic field (Fig. 7c) the slice chamber is closed. When applying the external magnetic field (Fig. 7a), the rings are arranged in the direction of the external magnetic field thereby opening the chamber for biopsy performance and approaching the intestine. Removal of the external magnetic field (Fig. 7b) results in taking a sample and placing it in the chamber (Fig. 7c). Modifications of the described method apply to triggering the moment of sampling—activating the knife (knives). In addition to the discussed method which uses a magnetic field, it is also possible to take advantage of the energy stored in the spring—Fig. 8. Figure 8 shows sampling of an intestinal slice using an open chamber with a strained spring and then releasing the spring while taking the sample and securing it in the chamber [76]. The sample size is about 1 mm^3^ while the biopsy chamber in the described cases constitutes approximately 2, 3 mm of the capsule length. A significant problem that is currently being solved is that the site is not visible while sampling. There exist known solutions [77] in which the camera is placed in the capsule axis (as in Fig. 9) partly enabling visualization of the biopsy site. There are also solutions which involve the use of micro handles that are emptied from a special capsule tank. The grippers are made of metal with shape memory. Owing to the intestinal temperature, when they touch its wall they grip an intestinal slice and cut it. Micro grippers have a size of about 1 × 2×1 mm and are collected by the same capsule using adhesion forces [78]. The block diagram of this solution is shown in Fig. 10. Withdrawing micro grippers (using adhesion and cones) is not in this case too effective. With an external magnetic field controlling the capsule, the effectiveness is about 22 %, and with no external electromagnetic field it is only 3 % [78]. In addition, the disadvantage of this solution is placing the camera on the opposite side relative to the cones which are designed to withdraw micro grippers. Nevertheless, it is one of the most interesting solutions proposed in recent years. These solutions, however, are still at the stage of clinical trials.

### Controlling the capsule movement

**Fig. 11:**
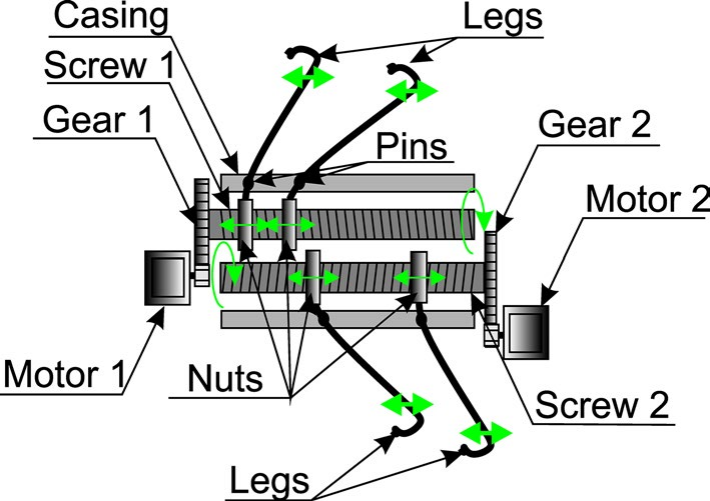
Block diagram of the self-propelled capsule fitted with two sets of legs driven with two separate gear motors. The two motors which work independently drive threaded rods by means of the gears. These in turn allow for the movement of two sections of legs along the capsule. Depending on the number of legs, various parameters are obtained, which are associated with the arrangement of the intestine around the capsule and, therefore, its invasiveness and the possibility of the intestine perforation

The possibility of controlling the capsule movement is much more difficult technically. In this area, there are two known solutions which are being tested. One of them is related to placing servo-controlled legs, 4, 6 or 8 legs, depending on the solution, ensure the capsule movement speed of 0.5 cm/min in both directions [79]. Figure 11 shows the block diagram of such a capsule. Controlling 2 independent leg assemblies is performed by using two independent actuators which include: a motor, gear, threaded rod, set of nuts, set of pins and legs (Fig. 11). It should be noted, however, that this solution does not contain important additional elements such as a camera or even a set of tanks for the drug.

**Fig. 12:**
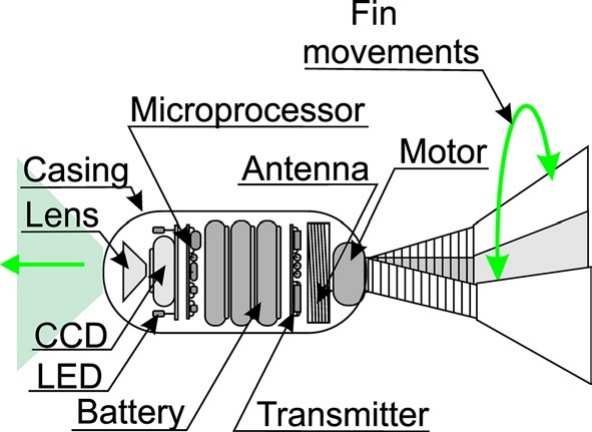
Schematic diagram of the capsule with a drive in the form of fins. The motor placed at the end of the capsule drives the tail causing swinging movement. Both the frequency of movements and amplitude can be adjusted using the embedded microprocessor. The other elements, including the camera are placed in the usual way in the main axis of the capsule

The second solution involves placing motorized fins at the end of the capsule—Fig. 12 (Japanese Universities). However, such a solution (Fig. 12) has numerous drawbacks. The biggest drawback is the introduction of a mobile element (fin) that has a direct contact with the surface (intestine). The fin size, depending on the solution, is about 10 × 45 mm. The obtained movement speed is 1 cm/s.

**Fig. 13:**
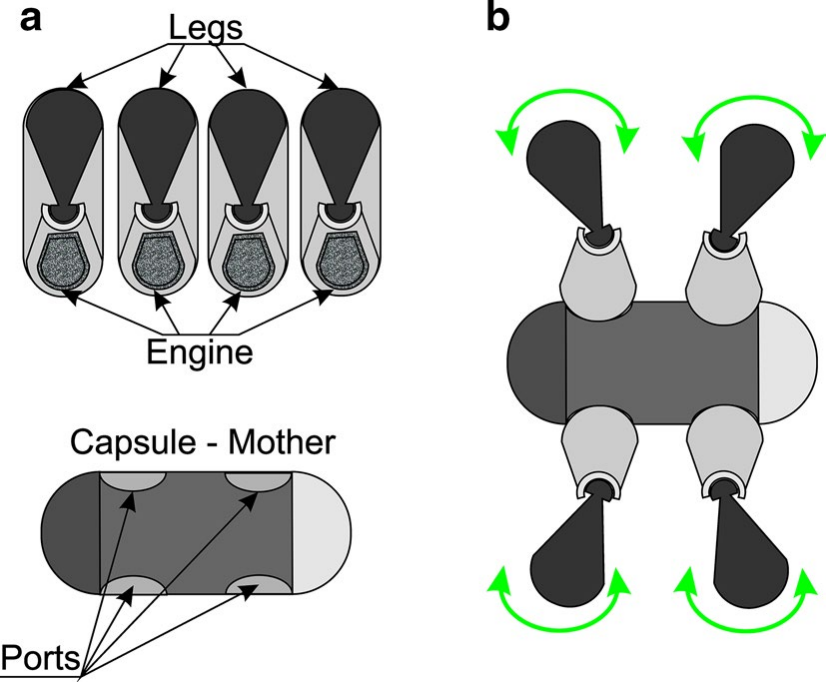
Block diagram of **a** components of the robot constructed of five capsules, **b** the robot assembled inside the human body. The robot includes a “capsule mother” allowing for reconfiguration and connection of individual parts of the robot inside the human body. The patient swallows not one but a few capsules (parts of the robot), which are then combined and allow for various tasks in the human body (monitoring, biopsy, movement and others)

A different type of solution is represented by reconfigurable robots consisting of several capsules proposed in 2013 by Rama, Jason Pui, Ben Szewczyk and Yoo Seung-Schik from Boston University. A patient swallows a few capsules that reconfigure in the patient’s abdomen and are able to carry out any surgery. Each robot is comprised of a central capsule to which movable legs are automatically attached—Fig. 13. Problems that occur here are associated primarily with the fact that the capsules need to find one another and join together in the human digestive tract. Situations known from other previous solutions, for example, the possibility of bowel perforation during movement, are equally problematic.

The presented solutions of endoscopic capsules constitute the prospects and expectations related to the initially mentioned problems. Two of the most important problems are associated with:

- movement in the intestine—using the external electromagnetic field (Fig. 5) or the capsule drive with the use of an external actuator (Figs. 11, 12);
- performance of biopsy: using an external electromagnetic field (Fig. 8) or a self-propelled capsule (Fig. 10).

Only the methods of capsule control using an external electromagnetic field described in the previous section have been used in clinical practice. The other solutions (Figs. 7, 8, 9, 10, 11, 12, 13) are still at the stage of clinical trials and are not widely available.

## Clinical usefulness, limitations and possibilities of increasing the scope of capsule endoscopy

The presented technical solutions are clinically useful and applied in varying degrees (as detailed previously). Most reports (99 % according to the Web of Science) refer to basic, most common types of endoscopic capsules (listed in Table 2). This also translates into the number of examined patients. For example, the number of patients examined with the use of capsule endoscopy by Given Imaging (PillCam: SB2, eso, colon) is 1.5 million to date, as declared by the company. The overview of Web of Science database for the year 2014 in the field of clinical reports enables to specify the information provided in Table 3.

**Table 3:** Summary of the number of analysed cases and types of diagnosed diseases in clinical publications published in 2014

Publication	Number of diagnosed cases	Type of disease	Results
Laurain A. [82]	80	Recurrent oesophageal varices	Capsule adequately classified 77.5 % of the patients for prophylaxis indication
Jeon S. [83]	2879	Cirrhosis patients with portal hypertension	The prevalence of PHE was 40 %. Comparing the PHE and non-PHE groups, the most common findings were angiodysplasias in 55.7 % and varices in 38.9 %. Active bleeding was observed in 16.6 and 3.7 %, respectively
Farnbacher M. [84]	65	Colorectal polyps	At a 30 % QV (QuickView) setting, the QV video presented 89 % of the significant polyps and 86 % of any polyps with ≥1 frame (per-polyp analysis) identified in NM before. At a 10 % QV setting, 98 % of the 52 patients with significant polyps could be identified (per-patient analysis) by QV video analysis
Ou G. [85]	6	Impact of chewing gum	Chewing gum did not have any significant effect on gastric transit time (rate ratio 1.06; p = 0.75), small-bowel transit time (rate ratio 0.91; p = 0.65), or completion rate of capsule endoscopies
Triantafyllou K. [86]	75	Complementing incomplete colonoscopy	One-third of the patients underwent capsule endoscopies immediately after colonoscopy
Oliva S. [87]	198	Small bowel cleansing by capsule endoscopy addressed in children	It supported the use of 25 ml/kg of polyethylene glycol solution plus 20 ml (376 mg) of oral simethicone as the preparation of choice for capsule endoscopy

The summary given in Table 3 shows that the total number of cases described in the clinical reports in 2014 exceeds 3000. These are mostly works in the field of research with a high level of detail, for example, analysis of the impact of the chewing gum [85] or diet for children [87] prior to the use of capsule endoscopy. However, in 2014 most works were published as reviews in the area of capsule endoscopy. These works can be divided into typical overviews of capsule endoscopy (works [89–92] and [99]); (2) profiled review papers. In the second area (profiled review papers) the works on the impact of wireless data transmission on the operation of cardiac pacemakers [93], or the detection of bleeding, [88] and [95], are of interest [96–98]. The overview presented in work [94] concerns the analysis of practical usefulness of one of the new PillCam options, namely automatic adjustment of the number of registered frames to the rate of changes in the content of the observed area. To conclude, the data summarized in Table 3 and other works that have appeared in the area of capsule endoscopy in 2015 [100, 101] show:

1. Detection of polyps >6 mm is at the level of 84–89 % (for comparison, colonoscopy—100 %).
2. Intestinal cleanliness, when using standard methods is at the level of 78–81 %.
3. Diagnosis of Crohn’s disease.
4. Identification of bleeding in the small intestine—effectiveness of 92.3, 44.2, and 12.9 % for patients with ongoing-overt bleeding, obscure-occult bleeding, and past bleeding.
5. Recurrent oesophageal varices—effectiveness of 70–80 %.

Undoubtedly, these great advantages of capsule endoscopy also have their limitations and resulting complications. These include:

- capsule blockage in the small intestine—about 0.75 % of all cases, 1.25 % of patients with Crohn’s disease [80, 81];
- problems with swallowing the capsule by the patient (in such cases the capsule is placed by means of endoscopy in the patient’s stomach);
- lack of research in the last sections of the intestine in patients with sluggish bowel motion and the associated maximum capsule operating time (8 h)—the problem occurs in about 33 % of patients;
- no capsule control—lack of possibility of seeing the same area again (in the simplest and most popular solutions—Table 2);
- no possibility of sampling—biopsy. New solutions, mentioned in the previous section, are still not common, or are in the process of research;
- inability to perform simple operations.

### New directions of research in the field of capsule endoscopy

Some of the above disadvantages of capsule endoscopy constitute a new current research area which includes:

- ensuring capsule mobility inside the intestine without an external electromagnetic field—preferably in both directions. Various capsule prototypes have been developed in this respect, but there are significant difficulties in miniaturization and approval for general use (4, 6, 8 legs driven by two independent servos, or the capsule developed in Japanese Universities—Fig. 12);
- enabling the performance of simple operations, sewing or drug delivery. As for simple operations, a module of biopsy (discussed in the previous section) has been developed. Drug delivery and pH measurement are available in a capsule (iPill solution developed at Philips Research Laboratories), but this solution does not contain a CCD camera;
- adjusting a proper diet, type of fluids administered to a patient before the examination in such a way so as to get the most accurate images without interference of food debris while ensuring the best possible comfort of the patient;
- automating the software, preferably to such a form in which the algorithm would separate the interesting area, and then compare with the base of standard pathologies and give hints to the doctor. The hint would automatically indicate which video part is the most interesting, and how large the change (pathology) is in terms of quantity (not quality);
- using other wavelengths in the visible range (possibly outside the visible range) and analysis of results in the form of, for example, hyperspectral analysis.

Another interesting research area is the examination cost reduction. Currently, due to the cost, it must be first decided whether capsule endoscopy is really necessary, and then, if the capsule endoscopy examination affects clinical decisions. In paper [102] in a study of 128 patients with suspected colorectal pathological lesions, the results of capsule endoscopy in 94 patients did not affect previous clinical decisions. At the same time the authors of paper [102] emphasized the validity of the examination in patients who were to undergo surgery. In work [103] it was found that in 26 % of patients (out of 300 examined) the result obtained from capsule endoscopy contributed to changing clinical measures. The group of patients included, in particular, those with unexplained bleeding. On the other hand, in work [104] the average cost of tests was analysed for 369 patients. It was found that the average cost of a positive diagnosis in patients who underwent capsule endoscopy was 2090.76 euros, and in those diagnosed by other methods—3828.83 euros. A reduction in the examination cost will be in the coming years a consequence of technological advancements, and thus miniaturization and universal access to endoscopic capsules.

### Restrictions on the use of capsule endoscopy

The technology and specifics of the capsule operation still limit the application of capsule endoscopy. In particular, these limitations include:

- problems with the local contamination of the capsule visual track. Despite doing the test under fasting conditions, local bubbles of fluids often prevent proper observation of the intestine [105]. The problem is too big to get rid of it completely. The only solution in these situations is to control the external magnetic field in order to clear the optical path or to omit local clusters of bubbles of fluids. However, it is possible to use an external magnetic field only in a medical facility, which increases the examination cost related to hospitalization and the need for continuous observation of the patient by a physician. At present, this problem is still not resolved;
- a problem with competing methods. Compared with competing methods, capsule endoscopy is used in a limited range of diagnostics, i.e.: in the diagnosis of unexplained bleeding and diagnosis of Crohn’s disease. It is less effective in the diagnosis of other diseases (other methods: single-balloon enteroscopy, spiral enteroscopy, double-balloon enteroscopy give better results) [106];
- a problem with proper preparation of the patient’s intestine for the test. This problem is related not only to the area of good vision of the camera mentioned before, but also to the responsibility of medical personnel for appropriate preparation of the patient for the examination (cleaning the intestine) and sometimes hospitalization (increase in the examination cost);
- no refund for this type of examination by the National Health Fund (NHF), the Food and Drug Administration (FDA). The availability of tests using capsule endoscopy in smaller towns in many EU countries and the USA is still limited [106];
- limited use. In fact, despite many solutions of capsule endoscopy presented above, it is still more or less accurate imaging (at various resolutions and with different numbers of cameras). There is no effective way to increase the range of use not only to enable biopsy of organs but also to perform any operations or even to stop the capsule in the intestine. Therefore, today endoscopy is still very limited to specific diagnostic purposes, basically one—detection of undisclosed bleeding from the small intestine [107];
- problems with the demonstration of statistically significant relevance and diagnostic sensitivity in detecting the small intestine or colon cancer [107]. This restriction is the reason why capsule endoscopy is not used interchangeably or even as a screening test in relation to classical colonoscopy [108];
- problems with ensuring economically justified further medical procedures (preparing the patient for the test again) after discovering irregularities in the patient’s intestine. After detecting bleeding or any other abnormalities of the gastrointestinal tract, the patient is hospitalized and subsequent stages of clinical procedures are implemented—the patient must be prepared for surgery again, a medical appointment must be arranged and so on. The total number of treatments and medical procedures is greater when using capsule endoscopy. The only economic profit and greater patient’s comfort are ensured when no irregularities are detected (lack of bleeding) [109];
- problems with limited operating time of capsules [110]. The operating time of 2–3 h is usually insufficient due to reduced intestinal motility. For this reason, the last section of the large intestine is not tested [111].

### Possibilities of increasing the scope of capsule endoscopy

Despite these drawbacks, it is possible in the near future to increase the scope of capsule endoscopy by:

- increasing the camera (cameras) resolution. This translates into not only more accurate visualization of the intestine but also the possibility of replacing (after appropriate treatment) damaged or non-valid (obscured by bubbles of fluids lingering in the intestine) nearest video frames;
- increasing the operating time of capsules by enhancing battery capacity and/or reducing the power consumed by the image recording system of the capsule;
- reducing the diameter of the endoscopic capsule and integrating it with the patency capsule. This will allow the dissolution of the outer layer of the capsule and leaving the intestine in the case of its blockage. In addition, integration will reduce the cost of the test (using one capsule instead of two);
- increasing the sophistication of image analysis and classification of diseases. This will enable not only a further reduction in the length of the video examined by a physician but also detection of new bowel diseases whose diagnosis is currently impossible—e.g. colorectal cancer. Probably it will require three-dimensional reconstruction of trajectory of the capsule movement as well as acquisition of additional data related to the capsule movement;
- increasing the accuracy of measurement of the intestine damage or the size of polyps. In this respect, calibration of the measurement path as well as image analysis and processing are probably sufficient. The new direction of the measurement path calibration might necessitate, for example, adding to the capsule a module for laser evaluation of its distance from the intestinal walls;
- providing a new type of housing allowing for multiple use of the same capsule. Then it would be possible to introduce cheaper diagnostics in less densely populated areas where access to capsule endoscopy is limited or even impossible due to high costs;
- providing e-diagnostics. Currently, almost none of the producers of capsule endoscopy mention the possibility of remote interpretation of the recorded video—e-medical service. Universal access to smart mobile devices (smartphones, iPads) would ensure automatic contact with the doctor on duty and transmission of the video or its selected interesting frames (with automatic preliminary analysis of the video performed by means of the smartphone).

According to the author, all of these modifications can be introduced on the market in the next few years. They will increase the effectiveness of detection of additional bowel diseases, reduce the cost of treatment and the time of data analysis by the physician.

## Conclusions

The current direction of research related to endoscopic capsules focuses on increasing their versatility and scope. In addition, it is important to reduce the burden on doctors analysing an endoscopic video. The following issues are also important here: (1) versatility in the reception of wireless data transmission from the capsule; (2) higher prevalence of capsules containing an internal non-volatile data memory; (3) reduction in the examination cost. The hope for capsule endoscopy is a new area of knowledge related to the methods of image analysis and processing as well as 3D modelling. Owing to the information about the image and the capsule position as well as human anatomical features, it is possible to perform fusion of data from different systems supporting the capsule operation. In particular, it can involve combining information about the capsule position with the acquired image and modelling a given organ—the intestine. This represents a new direction of research that already provides new diagnostic quality.

It now seems that endoscopic capsules which record the results directly in the internal flash memory (like CapsoCam SV-1) will gain the greatest popularity. If reading data did not require the use of an additional dock, in conjunction with the pilot capsule Agila, it would be a fully autonomous diagnostic kit (two capsules) that could be distributed in pharmacies. Remote diagnostics would be possible, for example, with the use of modern methods of medical telediagnostics. However, these solutions are still the future of capsule endoscopy. Still, given the advances in technology and miniaturization of the electronics, more sophisticated and miniaturized capsule endoscopy systems can be expected in the coming years. Technical possibilities of capsule endoscopy are constantly changing with the growth of technical progress and miniaturization of intelligent robots. The need to increase the accuracy of sensitivity and specificity in the detection of cancer and other diseases of the intestine still leaves an open area for microelectronics, electro-mechanics and computer science.

## Abbreviations

VCE: video capsule endoscopy
WVE: wireless video endoscopy
CE: capsule endoscopy
WCE: wireless capsule endoscopy
WVC: wireless video capsule
RFID: radio-frequency identification
